# Quality Control of Fried Pepper Oils Based on GC-MS Fingerprints and Chemometrics

**DOI:** 10.3390/foods14091624

**Published:** 2025-05-04

**Authors:** Jianlong Li, Yu Zhang, Qiang Cui, Zhiqing Zhang, Xiaoyan Hou

**Affiliations:** College of Food Science, Sichuan Agricultural University, Ya’an 625014, China

**Keywords:** *Zanthoxylum bungeanum* Maxim., *Zanthoxylum armatum* DC., fried pepper oils, volatile profiles, Similarity Evaluation System for the Chromatographic Fingerprint of Traditional Chinese Medicine

## Abstract

*Zanthoxylum bungeanum* Maxim. (huajiao) and *Zanthoxylum armatum* DC. (tengjiao), also known as Sichuan pepper, is a popular spice owing to its unique aroma and taste. Fried pepper oils are liquid condiments with unique flavors extracted from the pericarps of huajiao and tengjiao. To investigate the volatile profiles of the two different fried pepper oils, solid-phase microextraction (SPME) coupled with gas chromatography–mass spectrometry (GC-MS) was employed. The results revealed that D-limonene, linalyl acetate, linalool, myrcene, and ocimene significantly contributed to the overall flavor of huajiao oils. In addition, linalool, D-limonene, sabinene, myrcene, and linalyl acetate were identified as the main odorants in tengjiao oils. Finally, a characteristic chromatogram for the volatile compounds of each oil was established through the Similarity Evaluation System for the Chromatographic Fingerprint of Traditional Chinese Medicine, and the similarity thresholds of huajiao oils and tengjiao oils were 0.984 and 0.998, respectively. Linalool, sabinene, and linalyl acetate were markers for distinguishing between ZAOV samples and ZAOC samples. And germacrene D, linalool, sabinene, linalyl acetate, and β-myrcene were markers for distinguishing ZBOV samples from ZBOC samples.

## 1. Introduction

Sichuan pepper, also referred to as huajiao, is a shrub belonging to the *Zanthoxylum* genus in the Rutaceae family [1]. They have long been used as spices and in traditional Chinese medicine for the treatment of vomiting, toothache, stomachache, abdominal pain, and infected wounds [2,3,4]. Some bioactive extracts, such as essential oils, sanshools, and alkaloids, exert antimicrobial and antioxidant effects and regulate blood sugar levels [5,6]. *Zanthoxylum bungeanum* Maxim. and *Zanthoxylum armatum* DC. are two species extensively cultivated in Sichuan Province, China. “Hanyuan hua jiao” and “Hongya tengjiao” are popular varieties of *Z. bungeanum* Maxim. and *Z. armatum* DC., respectively. They have been recognized as geographical indication products of Hanyuan and Hongya because of their unique characteristics. The pericarp of mature Hanyuan huajiao is red and was historically called gong jiao because it was listed as a tribute during the Tang Dynasty. In contrast, Hongya tengjiao has a green pericarp. In addition to the different colors, Hanyuan huajiao and Hongya tengjiao have different flavors [7]. It is well documented that volatile components are the key quality indices of peppers. Freshly harvested peppers are highly seasonal, with a high moisture content and short storage period. If not treated in a timely manner after harvest, they are prone to wilting, browning, mold growth, and the deterioration of flavor quality. After harvest, fresh peppers are typically processed into dried pepper, pepper powder, and fried pepper oil.

Fried pepper oil is a popular condiment used in Sichuan cuisine. It is obtained by immersing hujiao granules in vegetable oil and frying them at high temperatures to extract unique flavor components. Compared with pepper granules, fried pepper oil not only has stronger flavor permeability, but also strengthens the salty taste [8]. Relevant studies have established that aldehydes, terpenes, alcohols, esters, and ketones are the primary aromatic components of fried pepper oils [9]. Meanwhile, compounds such as heptanal, nonanal, (E)-2-nonenal, and pentanal are primarily the products of lipid oxidation and significantly contribute to the fatty aroma of fried pepper oils. Terpenes, such as limonene, α-pinene, β-pinene, α-terpineol, and β-ocimene, are the most abundant substances in fried pepper oils and exhibit strong green, woody, and minor fragrances [10]. Lastly, alcohols such as α-terpineol and linalool predominantly impart a minty and herbal aroma to fried pepper oil, while esters and ketones principally contribute to fruity and floral fragrances [10].

However, the flavor profile of fried pepper oil is influenced by vegetable oil and raw materials. Thus, tocopherol, phytosterol, essential fatty acids, phenols, and other compounds present in vegetable oil, as well as the origin of huajiao and the production process, markedly affect the flavor of fried pepper oils. Therefore, ensuring product quality stability is a vital issue for factories that manufacture fried pepper oil. Fingerprinting techniques in conjunction with chemometric analysis have been extensively employed for the quality control of various products [11]. Chromatographic techniques such as high-performance liquid chromatography (HPLC), gas chromatography (GC), and thin-layer chromatography (TLC) coupled with detectors such as UV, DAD, and MS have been applied to develop chemical composition fingerprints [12,13]. Fingerprinting data combined with chemometric tools has the potential to assess the complex composition of a product. Pizarro et al. [14] utilized the GC-MS fingerprinting of volatile compounds to successfully differentiate olive oil samples from different geographical regions in Spain. Similarly, Shi et al. [11] pioneered an effective method for identifying adulteration in camellia oil by establishing a GC fingerprint of fatty acids and a GC-MS fingerprint of phytosterols in camellia oil.

Therefore, this study aimed to assess the use of the volatile profile of two different kinds fried pepper oils combined with the Similarity Evaluation System for the Chromatographic Fingerprint of Traditional Chinese Medicine for the quality control or differentiation of oil products produced by other companies. And the characteristic indicators used to distinguish different fried pepper oils were analyzed.

## 2. Materials and Methods

### 2.1. Materials and Chemicals

Forty different fried pepper oils, namely Hongya tengjiao oils (Brand A, named ZAO1–ZAO16 and ZAOV1–ZAOV4) and Hanyuan huajiao oils (Brand B, named ZBO1–ZBO16 and ZBOV1–ZBOV4), were procured from the supermarket of Hongya and Hanyuan County, Sichuan Province, China. Five competing products of tengjiao oil (ZAOC1–ZAOC5) and huajiao oil (ZBOC1–ZBOC5) from other brands were randomly collected from the local supermarket of Ya’an, County, Sichuan Province, China, for the verification test. N-tridecane (99%, Shanghai Macklin Biochemical Technology Co., Ltd., Shanghai, China) was used as an internal standard.

### 2.2. Extraction of Volatile Compounds from Fried Pepper Oils via HS-SPME

The volatile compounds of fried pepper oils were extracted using a headspace solid-phase microextraction (HS-SPME) method, outlined by Sun et al. [15] with minor modifications. Briefly, a fiber coated with 50/30 μm DVB/CAR/PDMS (Supelco, Bellefonte, PA, USA) was preconditioned for 30 min at 250 °C. Then, 5.0 g of fried pepper oil and 20 μL of n-tridecane (internal standard, 21.36 µg/µL in ethyl acetate) were mixed in a 20 mL headspace sample vial. Following this, the sample vial was placed in a thermostatic water bath at 70 °C. After equilibration for 15 min and absorption for 30 min, the fiber was inserted into the GC injection port for a 5 min desorption at 250 °C to perform the GC-MS analysis.

### 2.3. GC-MS Analysis

GC-MS analysis was performed using a 7890A GC equipped with a 5975C mass selective detector (Agilent Technologies, Foster City, CA, USA) according to a method described in previous studies [16]. All samples were analyzed on an HP-5MS capillary column (30 m × 0.25 mm × 0.25 μm). The carrier gas, helium, was delivered at a constant flow rate of 1 mL/min in hot splitless mode. The temperature of the injection port for GC was set at 250 °C. The oven temperature was initially held at 60 °C for 2 min, then increased to 160 °C at 4 °C/min and to 250 °C at 10 °C/min, and finally held for 5 min.

The volatiles were analyzed by MS in electron ionization mode with an electron energy of 70 eV, ion source temperature of 230 °C, and a mass scanning ranging between 40 and 550 *m*/*z*.

### 2.4. Qualitative and Quantitative Analysis

Qualitative analysis was performed by searching the MS data against the NIST14 mass spectrum database (National Institute of Standards and Technology, Gaithersburg, MD, USA). Volatile compounds were identified according to the matching degree and structural information of MS, and compounds with a matching degree exceeding 80 (Max. 100) were verified.

N-tridecane was introduced as an internal standard to semi-quantitatively assess the volatile compounds in fried pepper oils. The concentration of each compound was calculated using Formula (1):

(1) $$C_{i}=\frac{A_{i}}{A_{s}}\times C_{s}$$

where *C_i_* represents the concentration of the volatile compound (μg/g), *A_i_* denotes the chromatographic peak area of the volatile compound, *A_s_* is the chromatographic peak area of N-tridecane, and *C_s_* is the internal standard concentration in the sample (85.44 μg/g).

### 2.5. Methodology Validation

Methodology validation was conducted in accordance with Zhu et al. [17]. Briefly, the precision of the method was evaluated by replicating GC-MS injections of the same sample (ZAO 14) six times daily according to 2.2 and 2.3. The stability test was determined by injecting the same sample solution (ZAO 14) at 0, 4, 8, 12, 16, 20, and 24 h after preparation using a method according to 2.2 and 2.3. The repeatability was evaluated by detecting six copies of the same sample (ZAO 14) according to 2.2 and 2.3. Then, the relative standard deviation (RSD) of the retention time and peak area of common peaks were calculated.

### 2.6. Similarities Analysis

The similarities analysis was processed using professional software named Similarity Evaluation System for the Chromatographic Fingerprint of Traditional Chinese Medicine, composed by the Chinese Pharmacopoeia Committee (Version 2012A; Chines Pharmacopoeia Commission, Beijing, China). This software was used to calculate the correlation coefficients of the chromatographic profiles of 16 batches of ZAO/ZBO (48 chromatographic profiles in total) and the simulative mean chromatogram as a representative standard fingerprint/chromatogram (the reference fingerprint) was generated. The similarities of different chromatographic profiles were compared with the simulative mean chromatogram.

### 2.7. Application of Established GC-MS Fingerprints on Quality Control and Discrimination Products from Other Companies

Nine samples of each oil, including five with other brands (ZBOC1~ZBOC5, ZAOC1~ZAOC5) and four with the same brand (ZBOV1~ZBOV4 and ZAOV1~ZAOV4), were randomly selected on the local market. The volatiles were analyzed by GC-MS using a method described in 2.3. The similarity to the reference fingerprint was determined by introducing the GC-MS chromatogram to the Similarity Evaluation System for the Chromatographic Fingerprint of Traditional Chinese Medicine.

### 2.8. Statistics Analysis

#### 2.8.1. Hierarchical Cluster Analysis (HCA)

HCA of nine samples of huajiao oils (ZBOC1~ZBOC5 and ZBOV1~ZBOV4) or tengjiao oils (ZAOC1~ZAOC5 and ZAOV1~ZAOV4) was performed using Origin 2018 software. The essence of HCA is to simplify samples into different points in a multi-dimensional space, define the distance between the models using measurement methods, and divide the groups with similar properties into one class [18]. The same group has remarkable similarities, while different groups have significant differences. It is commonly used for unsupervised pattern recognition.

#### 2.8.2. Principal Component Analysis (PCA)

The contents of the common peaks of nine samples of huajiao oils (ZBOC1~ZBOC5 and ZBOV1~ZBOV4) or tengjiao oils (ZAOC1~ZAOC5 and ZAOV1~ZAOV4) were input to Origin 2018 software for PCA. The unit variance (UV) scaling was employed to perform PCA. PCs with cumulative percent variation (CPV) > 70.0–85.0% met the general requirement for PCA analysis and could fairly reflect most of the original data information [19].

#### 2.8.3. OPLS-DA Analysis

OPLS-DA was performed to analyze the GC-MS data more comprehensively to visualize the differences between different brands of huajiao oils or tengjiao oils using SIMCA 14.1. The most significant feature of this data analysis is its ability to remove the relationship between independent variable X and the independent change in the classification variable, thereby concentrating the classification information mainly on one principal component [20]. In the classification, the model becomes simple and easy to explain, and its discrimination effect and principal component, the visualization effect of the score chart, are more prominent.

## 3. Results and Discussion

### 3.1. Volatile Compounds of Huajiao Oils

A total of 41 types of volatile compounds (shown in Table S1) were identified in ZBO1–ZBO16, including alkenes (26), esters (4), alcohols (6), aldehydes (1), alkanes (2), and ketones (1). Among them, alkenes (with an average content of 594.83 μg/g) and alcohols (157.23 μg/g) played a key role in the volatile profile of huajiao oil, which is consistent with the findings of Ni et al. [8]. However, some oxygenated terpenes, such as citronellal and geranial, which have been identified as key odorants in ripe huajiao, were not detected in our study. The lack of these volatile compounds in our samples may be attributed to the different growth environments of huajiao [21], post-harvest treatments or processing methods. Moreover, our results suggested that D-limonene (297.64 μg/g, 28.91%), linalyl acetate (228.62 μg/g, 22.21%), linalool (146.138 μg/g, 14.20%), myrcene (130.25 μg/g, 12.65%), and ocimene (47.73 μg/g, 4.64%) accounted for 82.61% of the total flavor profile, significantly contributing to the overall flavor of huajiao oil. Notably, myrcene, limonene, linalool, and linalyl acetate have been identified as the main odorants in Hanyuan pepper oil [21,22]. Among them, limonene (297.64 μg/g) and myrcene (130.25 μg/g) impart a citrusy, lemony, and minty aroma to huajiao oil. Linalool (146.138 μg/g) contributes to a woody, sweet, and floral fragrance, while linalyl acetate (228.62 μg/g) offers a delicate and elegant scent similar to lily of the valley and lavender [23,24].

### 3.2. Volatile Compounds of Tengjiao Oils

As presented in Table S2, a total of 45 volatile flavor compounds were identified in ZAO1–ZAO16, including alkenes, alcohols, esters, aldehydes, and alkanes, with average contents of 948.02, 915.22, 64.68, 7.75, and 11.00 μg/g, respectively. The content of linalool (893.33 μg/g, 45.89%), D-limonene (361.67 μg/g, 18.58%), sabinene (214.22 μg/g, 11%), myrcene (108.90 μg/g, 5.59%) and linalyl acetate (47.73 μg/g, 4.64%) accounts for 81.06% of the total volatile flavor components, positioning them as the primary flavors of tengjiao oil. According to Liu et al. [25], linalool, β-myrcene, and limonene are the most potent odor-active compounds contributing to the overall volatile profile of ZAOs. Other important potent odorants included α-terpinene, ocimene, caryophyllene, and γ-terpinene. However, citronellal and geraniol, considered the most potent odor-active compounds, were not detected in the present study. The lack of these volatile compounds in our samples may be ascribed to the different growth environments of tengjiao, such as the illumination time and rainfall, which impact flavor characteristics [26]. Additional factors such as post-harvest treatments or processing methods can also influence the odor compounds in fried pepper oils. According to a prior investigation, linalool, myrcene, ocimene, and caryophyllene impart ZAOs with spicy, woody, and green notes [27], while limonene and α-phellandrene impart a citrus note. β-Pinene, terpinolene, and γ-terpinene impart ZAOs the pine and woody notes. Sabine contributed a turpentine-like flavor to ZAOs [25].

### 3.3. Identification of the Common Peaks

**Figure 1:**
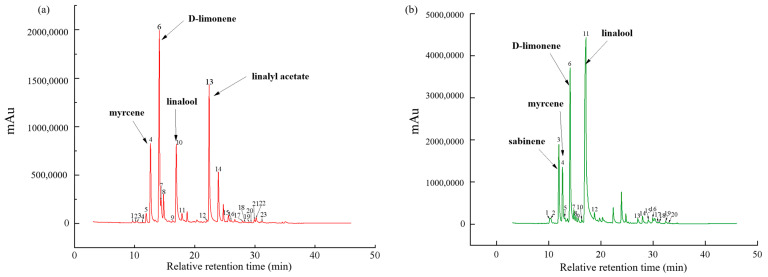
Standard chromatogram of volatile flavor components in ZBO (**a**) and ZAO (**b**). Different components are represented by numbers ranging from 1 to 20.

The GC-MS data of the huajiao oils tengjiao oils were imported into the Similarity Evaluation System to generate the fingerprint. As anticipated, consistent chromatographic peaks were observed in the volatile flavor components across all samples. The standard R1 and R2 fingerprints (Figure 1) of the volatile compounds in huajiao oil and tengjiao oil were automatically generated through multi-point calibration and automatic matching. As detailed in Table 1, a total of twenty-three common components were detected in huajiao oils, of which four were high in content, namely D-limonene, linalyl acetate, linalool and myrcene. At the same time, a total of 20 common peaks were detected in tengjiao oils, with high D-limonene, linalool, sabinene and myrcene concentrations.

### 3.4. Quality Evaluation of Fingerprints

Chromatographic fingerprints of herbs are a comprehensive qualitative approach to authentication and quality evaluation. The chromatographic profile should feature the fundamental attributes of ‘‘integrity’’ and ‘‘fuzziness’’ or ‘‘sameness’’ and ‘‘difference’’ so as to chemically represent the herbal medicines [28]. Considering these characteristics of fingerprints, the relative migration retention time and relative peak area of the main peaks detected were used to evaluate the quality of fingerprints.

The injection precision of the developed method was expressed as RSD. As shown in Table 2, the RSD of the relative retention time (RRT) and relative peak area (RPA) of common peaks did not exceed 2.85%. These results collectively indicate that the extraction and detection methods used in the experiment were reliable and effective.

### 3.5. Determination of Similarity

It is necessary for chromatographic fingerprints to be evaluated by their similarities, which come from the calculation of the correlative coefficient of the original data [29]. The similarity of the GC-MS chromatogram between different samples was obtained by the Similarity Evaluation System for the Chromatographic Fingerprint of Traditional Chinese Medicine. As summarized in Table 3, the similarity of the volatiles’ profiles among huajiao oils ranged between 0.971 and 1.000, while that for tengjiao oil ranged between 0.993 and 1.000. These results indicate that the chromatograms of samples with different production times were generally consistent and stable. The similarity of the volatiles’ profile in huajiao oil with the standard chromatogram R1 ranged from 0.984 to 1.000. For tengjiao oil (shown in Table 4), it is 0.998–1.000, indicating that it is easy to identify the quality of pepper oils based on this reference chromatographic. Therefore, 0.984 and 0.998 can be established as the similarity threshold of volatiles in huajiao oil and tengjiao oil, with values above 0.984 or 0.998 indicating that the products meet the qualified standards. Similar reports can be found in the quality evaluation of *Houttuyniae herba* [30], *Ganoderma lucidum* [29], and *Chaenomelis fructus* [17].

### 3.6. Discrimination of Different Fried Pepper Oils Based on Similarity

As detailed in Table 5 and Table 6, the similarity of volatiles between competing products and R1 and R2 were also compared. The results showed that all these competing products could be distinguished according to the similarity threshold of volatiles for huajiao oil (0.984) and tengjiao oil (0.998), with the exception of ZBOC2. Therefore, the volatile profiles of huajiao oil and tengjiao oil based on GC-MS can not only be used in quality control within the same brand products, but also be used for differentiating between different brands.

### 3.7. Hierarchical Clustering Analysis (HCA)

**Figure 2:**
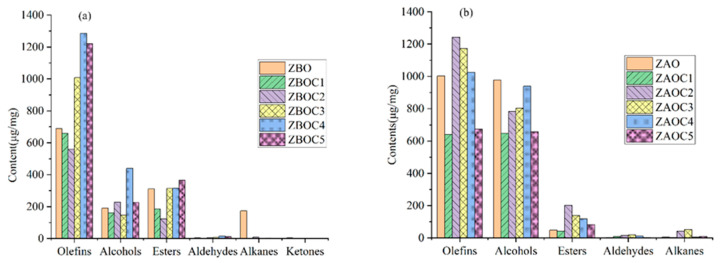
Types and contents of volatile compounds in ZBOs and ZAOs. (**a**) Contents of volatile compounds in ZBOs; (**b**) contents of volatile compounds in ZAOs.

**Figure 3:**
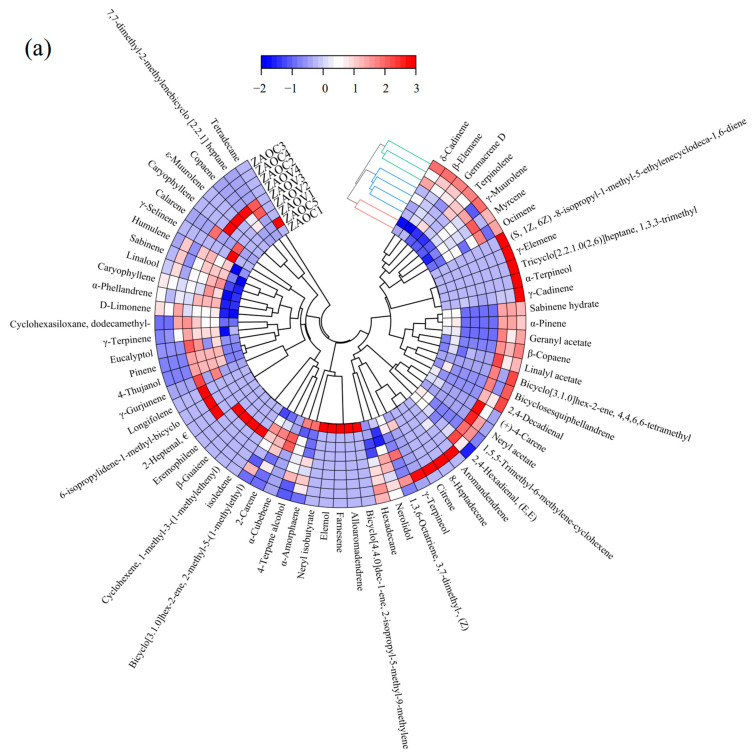

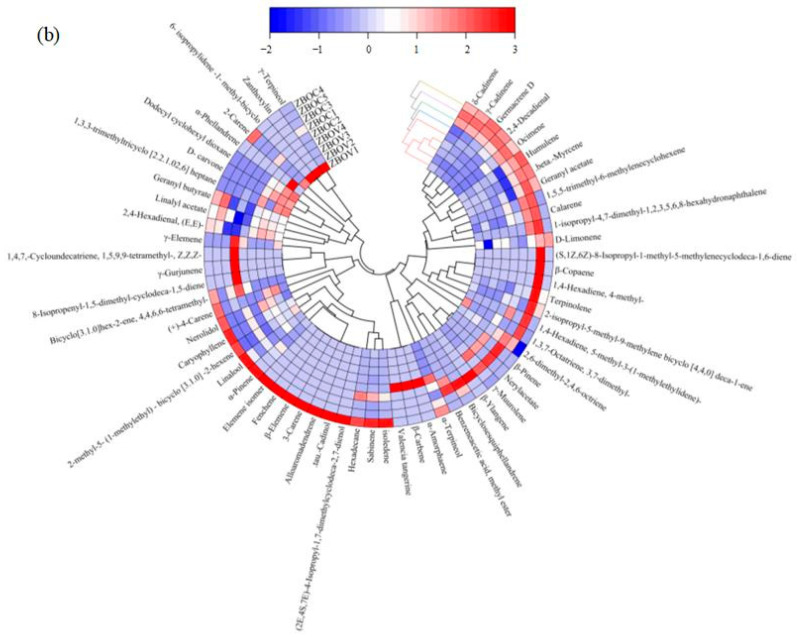
Clustering heat map of volatile compounds in ZAOs (**a**) and ZBOs (**b**).

HCA, a clustering technique, is largely used for grouping samples by delineating the hierarchy according to the similarities and dissimilarities among variables [31]. A total of 63 components were detected in ZBO (V1–V4) and ZBOC1–ZBOC5, including alkenes (43), alcohols (6), esters (5), alkanes (4), aldehydes (2), ketones (1) and others (2), among which 31, 30, 36, 25, 36, and 28 volatiles were present in ZBO (V1–V4), ZBOC1, ZBOC2, ZBOC3, ZBOC4 and ZBOC5, respectively. Interestingly, ketones were exclusively detected in ZBO (V1–V4). For tengjiao oils, a total of 67 compounds distributed in five classes were identified and quantified, of which 45, 33, 36, 35, 33, and 30 volatiles were present in ZAO (V1–V4), ZAOC1, ZAOC2, ZAOC3, ZAOC4, and ZAOC5, respectively. As illustrated in Figure 2a,b, the types and contents of these volatile components differed across brands. To visualize differences in the volatile profiles among different oils, an HCA heat map was generated, and the similarities between different samples were investigated. A system cluster analysis was performed using group average linkage methods and the Euclidean distance as the similarity. The results are displayed in Figure 3, wherein each volatile is represented by a colored box whose intensity is based on a normalized scale from a maximum of 3.0 (red) to a minimum of 2.0 (blue) to indicate the abundance of volatiles from high to low [32]. The clustering results uncovered three main clusters with similar characteristics. According to the composition and contents of volatile components, ZBOV1–ZBOV4 grouped together (shown in Figure 3b), distinguishing them from other samples, and ZBOC2 was more similar to ZBOVs, which is in line with the similarity comparison results. Likewise, as depicted in Figure 3a, samples could be divided into three categories for tengjiao oils, with ZAOV1–ZAOV4 being an independent group, which is in agreement with the similarity comparison results.

### 3.8. Principal Component Analysis (PCA)

**Figure 4:**
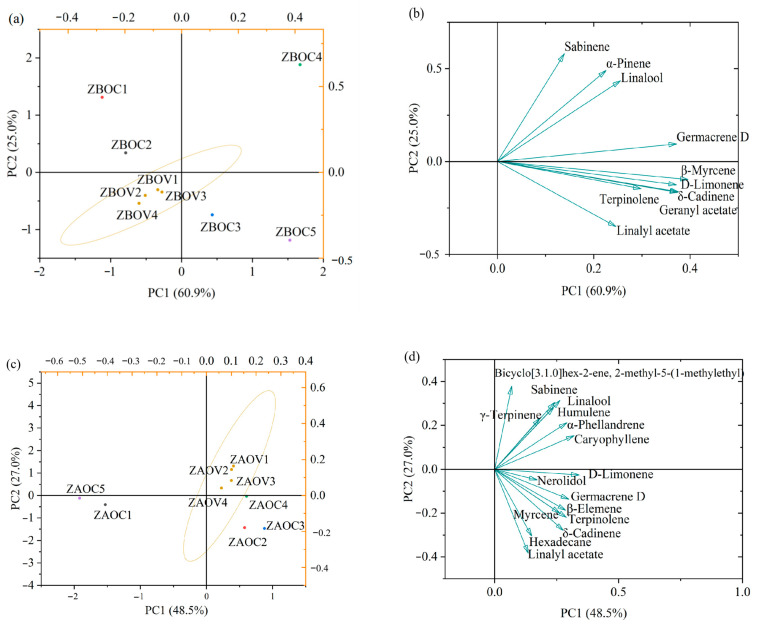
PCA clustering results of ZAOs and ZBOs. (**a**) Scores plot of ZBOs; (**b**) loading plot for common characteristic in ZBOs; (**c**) scores plot of ZAOs; (**d**) loading plot for common characteristic in ZAOs.

PCA is an unsupervised clustering method used to provide an overview of the variation in volatiles in samples without knowledge of the dataset [33]. The content data of common volatile compounds in ZAOs and ZBOs obtained by GC-MS were analyzed using PC in Origin software. Principal component analysis generated eight principal components for both ZBOs and ZAOs. In the case of ZBOs, the first two PCs (PC1 and PC2) explained 85.9% of the cumulative variance, while for ZAOs, PC1 and PC2 accounted for 75.4% of the total variance. Figure 4 illustrates the distribution of ZAOs and ZBOs in the orthogonal coordinate system of PC1 and PC2. As delineated in Figure 4b,d, common volatile compounds contributed differently to the classification of ZAOs and ZBOs, implying that the PCA models captured practical results. The farther the distance of volatile components from the coordinate origin, the more significant their role in distinguishing different fried pepper oils [29]. Figure 4a shows that ZBOs were divided into six clusters, of which ZBOVs (ZBOV1–ZBOV4) were classified into the same category while ZBOC2 was located closest to ZBOVs, indicating that ZBOVs share similar volatile characteristics and ZBOC2 was more similar to ZBOVs, in agreement with the HCA and similarity comparison results. Of note, as shown in Figure 4c, the scores of ZAOV samples clustered together in one group were separated from ZAOC2, ZAOC3 and ZAOC4 in PC2, while they were separated from ZAOC1 and ZAOC5 in PC1, consistent with the HCA results.

### 3.9. Orthogonal Partial Least Squares Discriminant Analysis (OPLS-DA)

**Figure 5:**
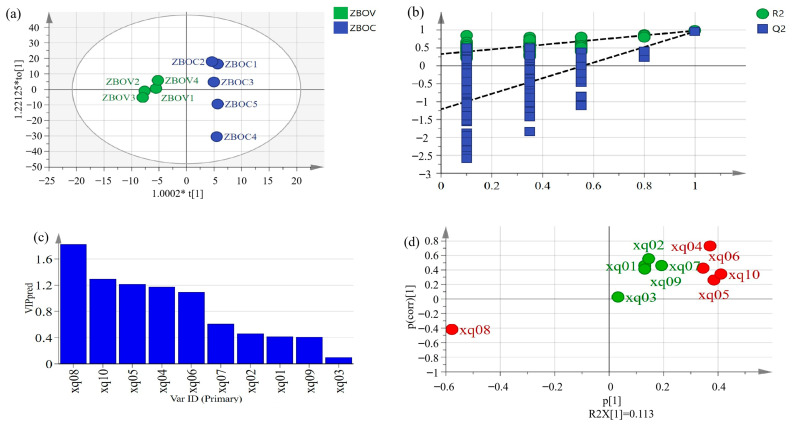
OPLS-DA model of ZBO samples. (**a**) Scatter plot of OPLS-DA of ZBOVs and ZBOCs. (**b**) 200 permutation tests for OPLS-DA model. (**c**) VIP value of different compounds in ZBOVs and ZBOCs. (**d**) S-plot of different compounds in ZBOVs and ZBOCs. (xq01: δ-Cadinene; xq02: α-Pinene; xq03: D-Limonene; xq04: Germacrene D; xq05: Linalool; xq06: Sabinene; xq07: Terpinolene; xq08: Linalyl acetate; xq09: Geranyl acetate; xq10: β-myrcene). The red dots indicate odors with VIP values exceeding 1, and the green dots indicate odors with VIP values not exceeding 1.

**Figure 6:**
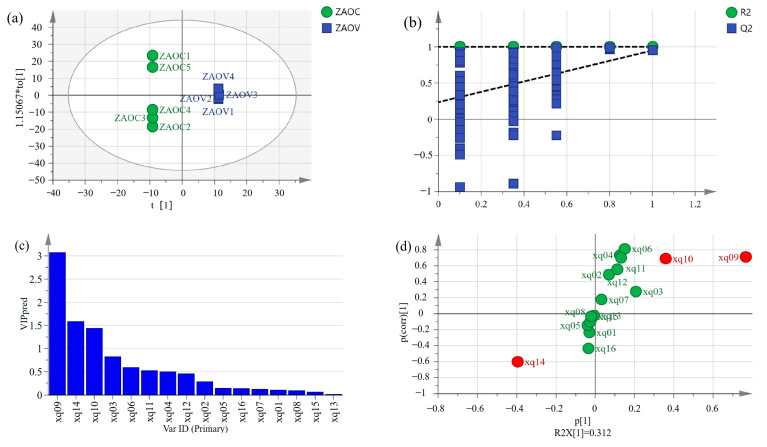
OPLS-DA model of ZAO samples. (**a**) Scatter plot of OPLS-DA of ZAOVs and ZAOCs. (**b**) 200 permutation tests for OPLS-DA model. (**c**) VIP value of different compounds in ZAOVs and ZAOCs. (**d**) S-plot of different compounds in ZAOVs and ZAOCs (xq01: δ-Cadinene; xq02: Bicyclo[3.1.0]hex-2-ene, 2-methyl-5-(1-methylethyl); xq03: D-Limonene; xq04: α-Phellandrene; xq05: β-Elemene; xq06: γ-Terpinene; xq07: Nerolidol; xq08: Germacrene D; xq09: Linalool; xq10: Sabinene; xq11: Humulene; xq12: Caryophyllene; xq13: Terpinolene; xq14: Linalyl acetate; xq15: Myrcene; xq16: Hexadecane). The red dots indicate odors with VIP values exceeding 1, and the green dots indicate odors with VIP values not exceeding 1.

OPLS-DA A is a supervised discriminant method widely used for quality evaluation. In the classification, the model becomes simple and easy to explain, and its discrimination effect and principal component, the visualization effect of the score chart, are prominent [20]. Figure 5 and Figure 6 show the OPLS-DA score scatter plots for ZBO and ZAO samples. Nine samples were clearly distinguished according to their different characteristics and most ZBOV and ZAOC samples were in the negative quadrant of t [1], while most ZBOC and ZAOV samples were in the positive quadrant of t [1]. The classification results are homologous to those of the PCA results in Figure 4. All results indicated that this OPLS-DA model has good stability and the ability to distinguish two kinds of pepper oil.

The variable important for the project (VIP) values were analyzed to determine the aroma components contributing greatly to fried pepper oils. VIP is the weight value of the OPLS-DA model variable that can measure the influence intensity and interpretation of the difference in accumulation for each component for the classification and discrimination of each sample group. The contribution rate increases proportionally with the VIP value. The greater the VIP value, the more significant the difference in variables among different varieties of fried pepper oils. Generally, VIP > 1 is the standard screening basis of differential components [34]. This study used VIP > 1 and *p* < 0.05 as the criteria. As Figure 5c illustrated, xq4 (germacrene D), xq5 (linalool), xq6 (sabinene), xq8 (linalyl acetate), and xq10 (β-myrcene) were selected by the VIP method as characteristic indicators for distinguishing between ZBOV samples and ZBOC samples. Additionally, xq9 (linalool), xq10 (sabinene), and xq14 (linalyl acetate) were markers for distinguishing between ZAOV samples and ZAOC samples.

S-plot was used to confirm the differences between different fried pepper oils and identify potential markers for distinguishing different samples, with the points at the ends of the “S” indicating the variables with the largest contributions to the model. In contrast, variables that contributed less were clustered around the origin. As shown in Figure 5d and Figure 6d, the red dots indicate odors with VIP values exceeding 1. Therefore, xq9 (linalool), xq10 (sabinene), and xq14 (linalyl acetate) were markers for distinguishing between ZAOV samples and ZAOC samples. The characteristic indicators for distinguishing ZBOV samples from ZBOC samples were xq4 (germacrene D), xq5 (linalool), xq6 (sabinene), xq8 (linalyl acetate), and xq10 (β-myrcene).

## 4. Conclusions

Herein, GC-MS coupled with the Similarity Evaluation System for the Chromatographic Fingerprint of Traditional Chinese Pharmacopoeia Committee was carried out to establish the volatile profiles of huajiao oils (Brand A) and tengjiao oils (Brand B). A total of 41 types of volatile compounds were identified in huajiao oils, including alkenes, esters, alcohols, aldehydes, alkanes, and ketones. Among those, D-limonene (297.64 μg/g), linalyl acetate (228.62 μg/g), linalool (146.138 μg/g), myrcene (130.25 μg/g), and ocimene (47.73 μg/g) accounted for 82.61% of the total flavor compounds, significantly contributing to the overall flavor of huajiao oils. At the same time, a total of 45 volatile flavor compounds were identified in tengjiao oils, including alkenes, alcohols, esters, aldehydes and alkanes, with linalool (893.33 μg/g), D-limonene (361.67 μg/g), sabinene (214.22 μg/g), myrcene (108.90 μg/g), and linalyl acetate (47.73 μg/g) identified as key odorants. Additionally, the similarity thresholds of huajiao oils (Brand A) and tengjiao oils (Brand B), as determined by the Similarity Evaluation System for the Chromatographic Fingerprint, were 0.984 and 0.998, respectively, which can be used for quality control. Linalool, sabinene, and linalyl acetate were markers for distinguishing between ZAOV samples and ZAOC samples. And germacrene D, linalool, sabinene, linalyl acetate, and β-myrcene were markers for distinguishing ZBOV samples from ZBOC samples.

## Figures and Tables

**Table 1:** Common peaks in 16 huajiao oils and tengjiao oils.

Huajiao Oils		Tengjiao Oils	
Common Peaks	Relative Retention Time (min, RRT)	Common Peaks	Relative Retention Time (min, RRT)
1	9.68	1	10.23
2	10.21	2	10.41
3	10.40	3	11.94
4	11.93	4	12.64
5	12.65	5	13.10
6	14.11	6	14.10
7	14.41	7	14.81
8	14.82	8	15.20
9	16.31	9	15.62
10	16.93	10	16.32
11	17.88	11	17.14
12	22.05	12	22.35
13	22.40	13	27.05
14	25.60	14	27.95
15	26.03	15	29.05
16	26.66	16	29.91
17	27.96	17	30.92
18	28.35	18	31.16
19	29.05	19	32.38
20	29.34	20	33.19
21	29.92		
22	30.94		
23	31.17		

**Table 2:** The precision, repeatability and stability of the method.

Peak No.	Precision (RSD, %)		Repeatability (RSD, %)		Stability (RSD, %)	
	RRT	RPA	RRT	RPA	RRT	RPA
1	1.81	0.32	1.05	0.28	0.99	0.35
2	1.66	0.66	2.21	0.33	1.45	0.27
3	1.58	0.34	1.98	0.56	1.88	0.36
4	1.33	0.32	1.66	0.92	2.01	0.15
5	2.56	0.21	1.88	0.88	2.13	0.22
6	2.62	0.69	2.05	0.52	1.77	0.37
7	1.88	0.36	1.96	0.49	2.05	0.29
8	2.79	0.29	1.85	0.66	2.13	0.48
9	1.92	0.38	1.66	1.02	1.97	0.55
10	1.45	0.41	1.79	0.76	1.96	0.29
11	2.06	0.36	1.91	0.68	2.45	0.37
12	2.33	0.55	2.05	0.81	2.85	0.36
13	1.96	0.38	2.11	0.91	2.46	0.39
14	1.46	0.62	1.91	0.53	2.11	0.41
15	1.88	0.56	1.88	0.68	1.98	0.32
16	2.76	0.76	1.67	0.99	2.03	0.26
17	2.08	0.55	2.06	0.76	2.66	0.33
18	2.11	0.62	2.13	0.62	2.25	0.51
19	1.99	0.39	1.98	0.77	2.20	0.33
20	2.33	0.31	2.14	0.72	2.08	0.27

**Table 3:** Similarity of volatile flavor substances in 16 ZBOs.

Similarity	ZBO1	ZBO2	ZBO3	ZBO4	ZBO5	ZBO6	ZBO7	ZBO8	ZBO9	ZBO10	ZBO11	ZBO12	ZBO13	ZBO14	ZBO15	ZBO16
ZBO1	1.000															
ZBO2	0.998	1.000														
ZBO3	0.998	0.999	1.000													
ZBO4	0.993	0.998	0.996	1.000												
ZBO5	0.995	0.998	0.998	0.998	1.000											
ZBO6	0.995	0.996	0.995	0.996	0.996	1.000										
ZBO7	0.978	0.979	0.977	0.980	0.979	0.986	1.000									
ZBO8	0.984	0.982	0.981	0.981	0.977	0.985	0.992	1.000								
ZBO9	0.971	0.976	0.972	0.981	0.975	0.981	0.985	0.981	1.000							
ZBO10	0.983	0.983	0.982	0.982	0.983	0.992	0.994	0.989	0.981	1.000						
ZBO11	0.989	0.988	0.989	0.987	0.988	0.994	0.992	0.993	0.982	0.998	1.000					
ZBO12	0.985	0.987	0.986	0.987	0.989	0.995	0.990	0.985	0.982	0.998	0.998	1.000				
ZBO13	0.992	0.995	0.994	0.994	0.996	0.998	0.986	0.982	0.981	0.993	0.995	0.997	1.000			
ZBO14	0.996	0.997	0.997	0.995	0.997	0.998	0.983	0.984	0.978	0.991	0.995	0.994	0.998	1.000		
ZBO15	0.993	0.995	0.995	0.994	0.997	0.998	0.985	0.983	0.980	0.993	0.995	0.997	1.000	0.999	1.000	
ZBO16	0.992	0.995	0.994	0.994	0.997	0.998	0.986	0.982	0.981	0.993	0.995	0.997	1.000	0.999	1.000	1.000
R1	0.995	0.998	0.996	0.998	0.997	0.999	0.989	0.989	0.984	0.992	0.995	0.994	0.998	0.998	0.998	0.998

**Table 4:** Similarity of volatile flavor substances in 16 ZAOs.

Similarity	ZAO1	ZAO2	ZAO3	ZAO4	ZAO5	ZAO6	ZAO7	ZAO8	ZAO9	ZAO10	ZAO11	ZAO12	ZAO13	ZAO14	ZAO15	ZAO16
ZAO1	1.000															
ZAO2	0.999	1.000														
ZAO3	0.999	0.999	1.000													
ZAO4	0.999	0.998	0.999	1.000												
ZAO5	0.998	0.998	0.998	0.999	1.000											
ZAO6	0.998	0.998	0.998	0.998	0.999	1.000										
ZAO7	0.998	0.997	0.997	0.997	0.998	0.998	1.000									
ZAO8	0.998	0.998	0.997	0.997	0.998	0.998	1.000	1.000								
ZAO9	0.998	0.998	0.997	0.997	0.998	0.998	0.999	0.999	1.000							
ZAO10	0.998	0.998	0.997	0.997	0.998	0.998	0.999	0.999	0.999	1.000						
ZAO11	0.998	0.998	0.997	0.997	0.998	0.998	0.999	0.999	0.999	1.000	1.000					
ZAO12	0.997	0.997	0.996	0.996	0.997	0.998	0.999	0.999	0.999	0.999	0.999	1.000				
ZAO13	0.997	0.997	0.996	0.996	0.997	0.998	0.999	0.999	0.998	0.998	0.998	0.998	1.000			
ZAO14	0.995	0.996	0.994	0.993	0.996	0.996	0.997	0.997	0.997	0.997	0.997	0.998	0.997	1.000		
ZAO15	0.996	0.997	0.995	0.995	0.997	0.998	0.997	0.998	0.997	0.998	0.998	0.999	0.997	0.999	1.000	
ZAO16	0.995	0.996	0.994	0.994	0.997	0.996	0.998	0.998	0.997	0.998	0.998	0.999	0.998	0.999	0.999	1.000
R2	0.999	0.999	0.998	0.998	0.999	0.999	0.999	1.000	0.999	0.999	0.999	0.999	0.999	0.998	0.999	0.998

**Table 5:** Similarity between ZBOCs and R1.

Samples	R1	ZBOC1	ZBOC2	ZBOC3	ZBOC4	ZBOC5
R1	1.000					
ZBOC1	0.958	1.000				
ZBOC2	0.987	0.976	1.000			
ZBOC3	0.946	0.983	0.962	1.000		
ZBOC4	0.982	0.961	0.991	0.929	1.000	
ZBOC5	0.980	0.944	0.987	0.915	0.997	1.000

**Table 6:** Similarity between ZAOCs and R2.

Samples	R2	ZAOC1	ZAOC2	ZAOC3	ZAOC4	ZAOC5
R2	1.000					
ZAOC1	0.996	1.000				
ZAOC2	0.989	0.991	1.000			
ZAOC3	0.996	0.995	0.993	1.000		
ZAOC4	0.997	0.995	0.991	0.998	1.000	
ZAOC5	0.983	0.983	0.998	0.988	0.987	1.000

## Data Availability

The original contributions presented in this study are included in the article/Supplementary Material. Further inquiries can be directed to the corresponding author.

## Supplementary Materials

The following supporting information can be downloaded at: https://www.mdpi.com/article/10.3390/foods14091624/s1, Table S1: Contents of volatile components in ZBO1~ZBO16; Table S2: Contents of volatile components in ZAO1~ZAO16.

## Abbreviations

The following abbreviations are used in this manuscript:

HPLC	High-performance liquid chromatography
GC	Gas chromatography
TLC	Thin layer chromatography
SPME	Solid-phase microextraction
GC-MS	Gas chromatography–mass spectrometry
PCA	Principal component analysis
HCA	Hierarchical cluster analysis
SFDA	State Food and Drug Administration
SD	Standard deviation
RSD	Relative standard deviation
