# Supplementary material for: Quality Control of Fried Pepper Oils Based on GC-MS Fingerprints and Chemometrics
Source: Foods. 2025 May 4;14(9):1624. doi: 10.3390/foods14091624 (PMC12071422; doi:10.3390/foods14091624)
Supplement: Supplementary file 1 [file foods-14-01624-s001.zip › foods-3532501-supplementary.pdf]

Table S1 Contents of volatile components in ZBO1~ZBO16

Volatile	ZBO1	ZBO2	ZBO3	ZBO4	ZBO5	ZBO6	ZBO7	ZBO8	ZBO9	ZBO10	ZBO11	ZBO12	ZBO13	ZBO14	ZBO15	ZBO16
δ-Cadinene	6.36±0.57	5.15±0.19	3.62±0.76	2.98±0.8	5.19±1.65	5.19±0.92	5.82±1.39	5.47±0.41	5.31±0.58	6.97±1.64	7.17±0.31	6.82±0.52	5.59±0.1	8.27±0.53	7.43±1.45	8.61±0.92
(+)-4-Carene	12.96±	34.26±4.27	26.15±6.15	12.45±2.84	21.31±4.26	27.18±4.14	14.92±3.14	26.32±1.67	33.02±3.86	28.93±4.78	14.38±2.51	36.08±5.49	15.78±1.72	39.99±0.79	37.5±1.18	21.75±0.88
Citrene	-	-	-	3.37±0.17	-	-	0.52±0.26	-	-	-	-	-	-	-	0.33±0.07	-
(E)-2-Heptene aldehyde	-	-	-	-	0.59±0.23	0.40±0.04	0.55±0.09	0.53±0.24	-	-	-	-	-	-	-	-
2,4-Hexadienal, (E,E)-	6.21±0.81	2.63±0.23	2.78±0.61	2.25±0.38	3.72±0.51	2.67±0.18	4.45±2.09	3.23±0.61	3.21±0.42	3.04±0.43	3.02±0.06	3.13±0.21	2.54±0.08	2.88±0.12	2.3±0.11	2.39±0.18
1,3,7-Octatriene, 3,7-dimethyl-	59.85± 11.37	-	29.41±6.96	-	-	28.5±1.49	35.8±3.99	-	-	-	-	-	52.8±0.75	60.74±0.54	51.35±2.07	52.47±2.01
1,3,3-trimethyltricyclo [2.2.1.02,6] heptane	45.79± 8.88	-	6.23±1.45	-	-	-	-	-	8.18±1.12	40.7±5.04	9.06±0.21	8.52±0.52	37.2±0.15	45.03±0.42	37.63±1.6	38.33±1.35
2,6- dimethyl -2,4,6-octatriene	23.67± 4.09	16.12±2.14	11.87±2.73	10.15±2.16	14.92±2.11	16.8±2.07	14.71±2.61	17.72±2.71	20.2±2.48	18.53±0.94	19.84±0.46	19.65±0	20.81±0.52	16.08±0.75	-	-
2- methyl -5-(1- methyl ethyl)-bicyclo [3.1.0]-2-hexene	1.28±0.24	1.93±0.25	1.93±0.4	2.11±0.43	1.6±0.22	1.91±0.1	2.77±1.05	2.15±0.3	1.49±0.22	1.67±0.26	1.59±0.05	1.49±0.06	1.03±0.11	1.55±0.09	1.34±0.08	2.51±0.14
2-Carene	-	11.28±0.85	-	12.47±2.74	-	-	-	5.44±0.89	-	-	-	-	13.09±0.03	-	17.49±1.26	-
2- isopropyl -5- methyl -9-methylene bicyclo [4,4,0] decyl -1- ene	2.03±0.11	-	1.21±0.37	0.94±0.26	-	-	-	-	-	-	1.88±0.11	1.76±0.11	1.79±0.05	-	-	-
4-(1- methyl ethyl) -2-cyclohexene -1- one	-	-	2.77±0.6	3.53±0.78	-	-	3.78±0.77	3.07±0.66	-	-	-	-	-	-	3.25±0.08	-
4- terpene alcohol	-	-	-	-	-	2.91±0.28	3.81±0.5	3.25±1.22	-	-	-	3.62±0.88	-	-	-	-
5- methyl -3-(1-methylethylene) -1,4-hexadiene	-	5.44±0.64	-	-	-	-	5.74±1.3	-	-	5.49±0.89	-	5.17±0.37	-	-	-	-
7- (1-methylethylidene) -bicyclo [4.1.0] heptane	-	4.39±0.14	4.98±1.65	4.43±1.12	4.98±0.75	0.71±0.09	5.43±1.38	-	-	-	-	5.36±0.04	-	-	-	-
α- Pinene	1.67±0.35	2.66±0.31	2.36±0.63	2.9±0.47	2.77±0.5	4.08±0.23	6.06±1.91	4.88±0.89	3.11±0.49	3.44±0.31	3.39±0.1	3.19±0.04	1.21±0.13	1.34±0.11	1.38±0.02	1.46±0.05
D-Limonene	369.91± 71.3	282.82±34.45	233.84±55.85	190.26±36.94	228.64±32.93	210.16±12.2	258.32±24.06	230.33±38.3	295.28±45.23	330.15±38.02	323.91±6.13	305.44±10.17	341.33±0.9	303.52±16.66	268.58±8.36	282.55±12.89
D- carvone	4.99±0.61	3.12±0.32	6.48±1.42	8.7±2.04	5.94±1.26	2.43±0.04	3.4±0.6	3.05±0.52	2.3±0.31	2.69±0.35	2.81±0.12	2.63±0.24	6.62±0.13	8.9±0.15	6.79±0.17	7.13±0.49
α-Phellandrene	-	4.74±0.48	4.49±1.07	4.43±0.89	5.25±0.2	5.68±0.87	6.54±0.76	6.22±0.27	5.56±0.56	-	-	-	-	-	-	-
α-Terpineol	-	-	-	8.96±2.13	-	-	7.66±0.16	10.65±1.9	8.98±1.38	8.59±1.2	9.97±0.28	9.21±0.51	10.89±0.03	15.79±0.3	14.02±0.5	14.12±1.04
γ-Cadinene	1.78±0.12	1.44±0.07	1.29±0.39	-	1.73±0.49	1.55±0.27	1.77±0.41	1.78±0.39	1.76±0.19	2.26±0.29	2.18±0.08	2.02±0.09	2.52±0.14	2.59±0.35	2.79±0.3	-
γ-Elemene	-	-	-	-	-	3.03±0.79	-	-	-	4.95±0.41	4.85±0.14	-	-	-	-	-
γ-terpinene	-	7.82±1.07		5.59±1.12	6.22±1.16	6.61±0.49	9.12±1.71	7.62±1.31	9.24±1.09	-	-	-	-	-	-	-
γ-Terpineol	-	3.19±0.41	4.59±1.06	4.88±1.13	3.71±0.87	2.6±0.21	4.1±0.99	3.3±0.55	2.65±0.62	2.77±0.34	2.73±0.25	3.05±0.21	3.52±0.18	3.23±0.22		
γ-Muurolene	-	1.23±0.09	0.95±0.25	1.81±0.22	1.6±0.35	1.47±0.3	1.51±0.18	1.94±0.22	5.73±0.18	1.77±0.02	1.46±0.03	0.84±0.04	6.21±0.63	-	-	-
Calarene	-	2.89±0.35	3.07±0.96	3.12±0.85	3.87±0.98	3.48±0.89	3.65±0.37	0.39±0.07	0.58±0.05	0.56±0.06	3.95±0.71	4.49±0.63	0.92±0.18	-	-	-
Nerolidol	-	3.34±0.26	1.75±0.6	1.89±0.63	2.84±0.72	2.9±0.74	3.35±0.86	2.1±0.18	2.92±0.42	2.65±0.14		2.8±0.37	-	-	1.61±0.36	1.78±0.25
Germacrene D	4.83±0.04	5.23±0.25	4.32±1.25	2.93±0.73	5.2±1.14	4.67±0.68	5.25±1	5.23±1.04	5.87±0.8	7.41±0.7	0.61±0.1	7.3±0.3	6.66±0.39	6.21±0.03	9.37±0.31	9.14±1.19
Geranyl butyrate	7.49±0.76	5.26±0.44	5.14±1.18	4.66±1.02	5.98±1.32	4.97±0.23	6.3±0.88	5.76±0.98	5.73±0.77	6.78±0.73	6.82±0.38	6.09±0.26	6.42±0.11	10.4±0.26	9.11±0.76	9.4±0.41
Linalool	176.28± 31.05	136.14±16.96	118.55±26.54	108.31±24.4	147.43±22.67	87.81±5.8	111.84±9.72	98.56±15.66	125.95±18.41	141.21±18.72	139.65±3.77	129.22±7.78	172.88±0.22	189.87±5.53	140.67±5.67	147.07±8.37
Sabinene	11.3±2.39	24.46±3.07	17.16±4.08	23.09±4.05	17.87±2.59	28.28±2.05	36.25±4.78	32.99±5.38	19.33±3.11	21.77±2.41	21.21±0.62	19.89±0.38	9.65±0.02	7.3±0.27	10.38±0.44	10.79±0.4
Zanthoxylin	-	5.78±0.28	4.07±1.27	6.49±1.75	16.75±14.4	16.26±3.8	18.39±4.79	8.99±1.08	11.69±2	10.5±0.45	11.85±1.02	0.97±0.39	-	-	-	-
Ocimene	-	41.41±4.89	22.51±4.63	31.03±4.79	31.07±5.18	43.72±6.48	49.35±6.3	48.27±0.91	45.14±2.38	-	-	-	-	-	-	-
Humulene	2.26±0.12	-	1.52±0.42	1.2±0.34	-	1.75±0.32	2.05±0.65	1.97±0.41	1.84±0.31	2.41±0.38	2.34±0.16	2.1±0.17	2.1±0.17	2.57±0.03	2.79±0.38	2.91±0.26
Dodecyl cyclohexyl dioxane	48.14±	23.61±9.61	24.88±9.42	37.55±8.98	14.19±6.2	15.63±6.86	19.76±11.49	23.62±7.83	21.04±11.69	22.16±14.2	27.32±7.97	12.85±6.38	43.27±12.87	21.73±2.51	29.79±15.6	25.21±7.7
Caryophyllene	3.49±0.22	3.15±0.22	2.89±0.77	2.5±0.59	3.47±1.11	3.19±0.32	3.9±0.72	3.71±0.67	3.65±0.52	4.43±0.44	4.36±0.17	3.92±0.1	3.83±0.07	4.8±0.09	4.63±0.49	4.73±0.17
Terpinolene	5.29±0.74	4.93±1.17	2.39±0.48	5.13±1.07	2.46±0.34	5.47±0.91	2.74±0.09	4.67±0.3	3.77±0.89	6.31±1.55	3.31±0.49	5.44±0.89	6.3±0.71	6.22±0.14	5.48±0.31	6.64±0.03
Linalyl acetate	295.38± 50.66	208.08±21.53	188.77±38.7	161.32±34.78	204.64±40.61	157.25±5.73	198.23±13.98	178.88±28.68	209.23±30.33	238.84±32.71	236.98±3.55	219.31±16.9[illegible]	220.32±4.47	260.38±4.55	206.29±7.86	210.55±8.76
Terpinyl acetate	-	-	-	-	-	12.14±0.9	15.06±1.52	13.47±1.94	15.9±0.5	-	-	-	-	-	-	18.08±0.97
Geranyl acetate	8.75±1	6.18±0.5	5.87±1.38	5.42±1.34	7.1±1.29	5.7±0.42	7.28±0.95	6.8±1.2	6.93±0.93	8.23±1.01	8.12±0.12	7.52±0.35	7.24±0.19	11.16±0.28	9.31±0.61	9.64±0.6
Myrcene	171.15± 33.28	123.89±17.36	92.76±22.05	70.32±13.64	94.86±13.42	82.9±4.73	102.76±10.72	90.43±15.25	122.36±18.47	137.72±16.45	134.03±1.88	126.24±4.55	142.84±0.06	151.62±5.87	126.94±5.14	131.02±5.35

Table S2 Contents of volatile components in ZAO1~ZAO16

Volatile	ZAO1	ZAO2	ZAO3	ZAO4	ZAO5	ZAO6	ZAO7	ZAO8	ZAO9	ZAO10	ZAO11	ZAO12	ZAO13	ZAO14	ZAO15	ZAO16
2,4-Hexadienal,	-	2.36±0.11	-	-	0.77±0.01	0.73±0.00	0.66±0.02	1.71±0.02	0.96±0.03	-	-	-	-	-	-	-
Bicyclo[3.1.0]hex-2-ene, 2-methyl-5-(1-	6.40±0.35	7.02±0.11	6.77±0.21	6.54±0.77	7.83±1.06	7.37±0.19	6.75±0.72	7.1±0.17	6.28±0.35	9.56±0.33	9.9±0.43	8.55±1.22	10.72±2.21	11.16±0.26	10.6±0.83	10.95±0.30
α-Pinene	10.89±0.39	12.66±0.53	11.35±0.42	12.38±0.98	14.2±2.00	14.25±0.62	13.28±1.24	12.93±0.46	12.04±0.14	19.71±6.40	15.1±1.10	13.03±1.75	16.8±3.12	18.55±0.18	16.67±1.90	16.65±0.67
2-Heptenal	-	1.41±0.12	-	-	-	-	-	1.79±0.05	-	-	-	-	-	-	-	-
Sabinene	170.00±10.85	190.24±6.57	181.16±1.82	190.55±14.19	216.68±29.25	220.82±7.61	204.11±16.09	192.5±3.36	189.16±5.33	255.68±14	233.89±14.51	196.07±24.86	243.34±47.3	262.03±2.62	244.68±21.85	253.52±9.19
Myrcene	96.66±5.27	97.88±4.64	93.6±1.02	95.65±6.89	105.25±14.98	105.78±3.51	98.67±7.81	92.88±1.18	95.01±4.09	122.12±8.74	112.03±8.2	93.71±12.48	113.99±23.28	118.54±2.89	119.31±9.64	119.38±4.36
α-Phellandrene	11.43±0.71	9.4±0.29	9.49±0.13	10±0.87	10.32±1.33	10.5±0.34	10.13±0.78	8.78±0.13	7.7±0.21	11.34±0.53	9.92±0.84	8.85±1.16	13.8±2.87	12.59±0.39	11.2±0.86	10.88±0.26
2-Carene	6.84±0.39	8.04±0.15	7.1±0.10	8.47±0.51	11.25±1.36	7.77±0.28	8.75±1.84	8.69±0.19	10.9±0.7	11.1±0.47	11.48±0.73	7.47±0.93	9.77±1.84	11.60±0.38	11.80±1.13	12.65±0.39
D-Limonene	313.79±15.34	326.41±13.03	315.17±4.11	323.89±20.17	352.77±46.57	352.16±9.78	326.4±22.76	310.06±4.65	319.18±12.78	410.99±21.64	379.69±26.91	313.95±40.44	384.38±76.99	392.64±10.76	391.96±30.43	402.14±13.20
Ocimene	31.43±2.46	25.21±0.93	30.84±0.91	31.33±2.47	30.91±3.67	32.02±1.33	30.21±2.70	24.93±0.27	26.01±0.81	32.95±2.30	30.19±2.18	26.24±3.60	32±6.62	34.35±0.70	36.15±2.90	35.06±1.31
γ-Terpinene	15.35±2.63	12.45±0.38	14.76±1.02	14.77±1.28	14.46±1.43	14.95±0.63	14.13±1.32	13.25±0.17	11.77±0.35	18.04±1.71	16.51±1.16	14.53±2.00	15.99±3.22	16.36±0.15	15.97±1.57	16.16±0.48
4-Thujanol	20.29±2.19	21.47±0.69	21.03±0.77	21.95±1.67	22.42±1.6	23.19±1.09	21.66±2.04	20.36±0.21	18.84±0.28	29.66±2.23	26.91±1.76	23.5±3.22	25.59±4.84	25.18±0.22	25.25±2.74	25.38±1.00
Terpinolene	10.95±0.77	7.32±0.19	10.71±0.33	10.8±0.88	9.18±2.05	8.63±0.39	8.38±0.82	10.28±0.08	5.9±0.14	14.24±0.76	9.7±0.49	11.51±1.54	12.95±3.03	10.55±1.73	9.97±0.89	9.65±0.17
Tricyclo[2.2.1.0(2,6)] heptane, 1,3,3-	-	-	-	-	2.34±0.38	-	-	-	2.24±0.06	-	-	-	-	2.66±0.29	2.74±0.18	2.79±0.03
Linalool	753.95±29.57	774.27±32.52	743.45±6.11	769.82±48.39	848.37±71.82	829.86±27.29	776.13±61.99	785.96±8.72	771.42±25.65	1138.65±74.29	1015.01±66.60	855.59±113.1[illegible]	1022.37±198.62	1071.13±10.81	1029.86±107.02	1051.84±38.78
4-Terpene alcohol		17.11±0.69	15.96±0.24	17.07±1.08	19.41±0.68	18.55±0.99	17.37±1.79	21.00±0.27	17.69±0.1	28.45±1.96	27.22±1.41	22.92±2.92	-	23.11±0.07	-	-
Cyclohexene, 1-methyl-3-(1-	-	-	-	-	-	-	18.06±0.58	-	-	42±17.12	-	-	-	-	-	-
γ-Terpineol	5.22±0.16				8.14±0.97		-	-	-	-	-	-	7.77±1.44	-	-	-
Linalyl acetate	46.57±2.17	59.91±3.02	57.41±1.37	60.5±3.03	69.23±3.46	70.47±3.11	66.52±5.88	57.56±0.46	62.9±1.26	82.18±5.96	80.84±4.32	64.29±7.25	79.69±16.07	74.72±0.31	74.14±9.39	69.66±2.72
	-	-	-	-	-	-	-	-	-	-	-	10.14±1.67	-	-	12.9±1.58	12.07±0.66
7,7-dimethyl-2-methylenebicyclo [2.2.1] heptane	2.33±0.29	3.01±0.16	-	-	3.51±0.30	3.58±0.25	3.32±0.13	3.71±0.08	-	-	-	-	4.56±0.81	4.53±0.08	4.47±0.67	5.25±0.07
2,4-Decadienal	-	4.74±0.16	5.87±0.26	-	6.61±0.68	7.02±0.43	6.88±0.55	7.19±0.32	5.88±0.28	7.55±0.2	8±0.1	7.38±0.35	7.24±0.39	6.90±0.79	7.97±0.18	4.74±0.16
1,5,5-Trimethyl-6-methylene-	5.72±0.11	5.23±0.16	5.56±0.14	5.59±0.31	6.17±0.50	6.45±0.33	6.22±0.21	6.15±0.21	4.62±0.27	7.57±0.62	7.39±0.13	6.17±0.4	8±1.21	8.48±0.58	8.47±0.87	9.55±0.16
α-Cubebene	3.81±0.11	3.81±0.18	4.18±0.04	4.41±0.34	-	5.01±0.03	4.67±0.34	4.68±0.23	3.96±0.14	7.3±0.89	7.67±0.37	5.95±0.29	6.39±1.13	6.33±0.33	7.14±0.72	7.3±0.26
β-Copaene	1.20±0.19	1.04±0.13	1.19±0.12	1.26±0.07	2.58±1.04	-	-	-	-	-	-	1.84±0.05	1.8±0.75	1.92±0.34		2.23±0.09
Geranyl acetate	-	-	-	-	-	2.83±0.14	2.8±0.04	-	2.64±0.50	-	-	-	4.33±1.54	3.68±0.01	5.62±1.19	3.63±1.05
β-Elemene	15.51±0.65	14.27±0.95	14.37±0.34	14.91±0.99	13.16±4.04	10.97±4.46	10.87±5.64	10.78±5.00	8.79±3.44	14.7±8.28	9.73±0.23	8.10±0.47	9.66±1.74	12.41±6.11	25.42±2.89	31.03±0.99
Tetradecane	-	-	-	-	-	9.17±0.16	8.56±0.54	8.67±0.63	7.25±0.24	12.16±0.15	12.91±0.31	10.31±0.43	11.41±1.98	11.21±0.80		
Caryophyllene	16.30±3.1	21.44±4.38	19.01±3.75	19.91±2.97	24.13±3.19	20.3±1.06	19.48±1.31	18.66±0.43	19.66±2.97	27.98±8.43	23.24±0.6	20.02±1.26	23.28±3.77	28.36±4.71	24.22±3.14	26.53±1.04
β-Copaene	6.16±0.14	-	6.22±0.11	6.75±0.17	-	7.25±0.39	7.09±0.45	6.67±0.31	-	8.82±0.39	8.85±0.26	7.61±0.35	9.14±1.51	-	9.82±1.14	11.17±0.43
γ-Muurolene	1.95±0.31	1.75±0.09	1.99±0.16	2.18±0.24	2.24±0.19	2.50±0.22	2.42±0.19	2.25±0.19	1.48±0.22	3.26±0.33	3.28±0.39	2.76±0.21	3.5±0.54	3.6±0.46	3.63±0.49	3.95±0.2
Humulene	11.36±0.22	15.38±1.08	15.85±0.46	16.41±1.31	17.15±1.83	18.75±0.99	18.16±0.82	17.26±0.73	14.23±1.18	22.52±1.9	21.93±0.43	17.96±0.67	15.36±2.59	18.32±3.36	14.91±1.88	16.81±0.73
Bicyclo[4.4.0]dec-1-ene, 2-isopropyl-5-methyl-9-methylene	4.58±0.49	-	-	-	-	-	-	-	-	-	-	-	6.41±1.11	-	6.41±0.51	7.68±0.21
Germacrene D	16.98±0.27	15.62±1.13	17.09±0.36	18.37±1.35	18.74±2.12	20.95±1.44	20.64±1.32	18.72±0.95	14.27±0.93	25.18±2.10	23.56±0.13	20.5±0.91	26.42±4.58	29.35±2.21	28.88±3.5	33.59±1.13
Alloaromadendrene	-	-	-	16.25±0.02	-	-	-	21.54±5.07	-	25.53±1.52	25.07±0.35	19.57±1.8	28.76±4.75	26.72±1.21	28.31±3.28	34±0.90
γ-Terpinene	3.11±0.61	2.58±0.16	3.02±0.19	3.09±0.4	3.12±0.42	3.60±0.18	3.47±0.14	3.23±0.3	2.31±0.22	3.93±0.62	4.03±0.07	2.50±0.91	4.37±0.79	4.56±0.3	4.32±0.38	5.09±0.3
δ-Cadinene	5.26±0.95	4.4±0.76	5.33±0.37	5.08±0.66	5.47±0.92	6.23±1.26	6.04±0.71	6.34±1.2	4.07±0.19	7.63±2.03	8.14±1.25	14.18±9.43	7.1±1.36	8.03±1.29	7.23±0.65	8.4±0.29
isoledene	1.50±0.22	0.9±0.24	1.36±0.01	1.46±0.09	-	1.56±0.04	1.56±0.05	-	-	-	-	3.94±1.25	2.25±0.43	2.43±0.21	2.35±0.23	2.74±0.15
Calarene	-	0.99±0.12	1.41±0.26	1.51±0.36	1.38±0.36	1.74±0.14		1.64±0.21	0.99±0.13	2.04±0.37	2.24±0.15	4.12±2.08	2.26±0.46	-	1.8±0.09	2.28±0.22
Nerolidol	12.39±1.71	8.97±0.5	12.95±2.28	11.82±2.77	12.81±3.55	15.15±0.68	16.16±1.71	11.19±1.69	9.23±2.24	13.83±0.77	13.97±0.64	1.79±0.17	16.9±5.7	16.19±2.54	12.91±0.44	16.74±1.92
Eucalyptol	-	-	-	-	-	-	-	-	-	-	-	-	-	-	-	-
Hexadecane	3.67±0.51	2.37±0.22	2.9±0.5	3.21±0.66	3.04±0.84	4.02±0.19	3.84±0.36	3.51±0.47	2.60±0.39	4.38±0.37	4.87±0.23	4.29±4.76	3.74±1.12	3.56±0.60	3.38±0.14	4.64±0.40
Aromandendrene	0.83±0.11	0.3±0.10	0.76±0.10	0.56±0.22	-	0.78±0.13	0.73±0.06	0.71±0.17	0.26±0.02	-	-	-	-	-	-	-
Longifolene	-	0.18±0.14	-	0.38±0.01	0.31±0.07	0.39±0.02	0.37±0.09	0.31±0.07	-	-	-	-	-	-	-	-
γ-Elemene	0.64±0.01	0.28±0.08	0.47±0.11	0.52±0.12	0.45±0.13	0.65±0.06	0.60±0.06	0.59±0.10	0.33±0.09	0.80±0.25	0.75±0.12	7.48±3.65	0.59±0.23	0.53±0.08	0.49±0.02	0.72±0.10
